# Socioeconomic differences in digital inequality among Chinese older adults: Results from a nationally representative sample

**DOI:** 10.1371/journal.pone.0300433

**Published:** 2024-04-02

**Authors:** Hongchao Hu, Wenqian Xu

**Affiliations:** 1 School of Humanities and Social Sciences, North China Electric Power University, Beijing, China; 2 Department of Health Sciences, Lund University, Lund, Sweden; 3 School of Social Development and Public Policy, Fudan University, Shanghai, China; BRAC Business School, BRAC University, BANGLADESH

## Abstract

The current study seeks to investigate digital inequality among older adults in China, specifically examining two socially defined age groups: young-old adults (aged 60–74) and old-old adults (aged 75+). Descriptive statistics and multiple regression were used to examine the prevalence of and identify the factors associated with Internet access, usage (frequency and breadth containing 11 activities), skills, and social support. The study used data from the 2018 China Longitudinal Ageing Social Survey (CLASS) which consisted of 11,419 respondents aged 60 years and older. We found that 40.22% of older adults had access to the Internet, and 18.27% used it regularly. Socioeconomic factors played a crucial role in determining Internet access and usage, with young-old adults with higher education using the Internet more frequently, deliberately, and competently. Those with higher economic status had greater social support to use it, and the old-old adults with higher socioeconomic status were more likely to have Internet access. This study has implications for prioritizing targeted policies and interventions aimed at supporting socioeconomically disadvantaged older adults and ensuring equal opportunities for all to access and benefit from the digital world.

## Introduction

With the increasing digitization of society, there has been a growing adoption of the Internet and technology among older adults worldwide in recent decades [1–3]. The active involvement of older adults in a fully integrated digital society is crucial, as this enables them to uphold their independence, engage in social activities, and participate productively in the evolving world [4, 5]. However, there are concerns about digital exclusion in old age as digital inequalities have been linked to negative outcomes for health and well-being [6]. The present study aims to examine digital inequality experienced by older adults in China, an issue that has received limited attention in the Chinese research community.

Existing literature highlights disparities in digital access and utilization among older adults, which have been described as a digital divide between young and older generations [7, 8]. While digital inequality is a widely studied issue in high-income countries, it is under-investigated among older adults in low and middle-income countries, including China. Research shows that older adults are less likely to use the Internet and digital technology than their younger counterparts, with certain subgroups of older adults being more susceptible to digital exclusion. Digital inequality encompasses various dimensions, such as access to equipment, independence of usage, skills, purpose of technology use, and availability of social support [9]. Current research on digital inequality goes beyond access disparities and encompasses inequalities among those who have access to digital technologies, including disparities in digital use, skills, and outcomes [8, 10, 11].

Multiple mechanisms for older adults’ technology adoption and usage are evident in existing digital inequality literature. When examining technology adoption and usage among older adults within the context of digital inequality, it is crucial to consider their socioeconomic background (e.g. age, income, education, and marital status) and availability of access points [10]. Furthermore, individual factors (e.g. purposes for use, digital skills, and behaviour control) [12] and social contexts (e.g. learning environments, the presence of social actors, and the support network around them) [13] must be taken into consideration, alongside the design of technologies.

Older adults are a diverse demographic group, encompassing individuals with varying socioeconomic and health statuses, pursuing different inspirations and interests, and having undergone distinct life trajectories that result in diverse health and social outcomes. In the realm of cultural gerontology, older adults are classified into two distinct age groups: the young-old (often referred to as third-agers) [14] and the old-old adults (fourth-agers) [15]. These age groups are socially constructed and culturally represented across different societies [16]. Young-old adults are typically perceived as joyful with ample opportunities, while old-old adults are often associated with negative attributes such as incapacity, poverty, and frailty. Positive ageism may be directed towards young-old adults who are not able to meet social expectations and cultural norms for active ageing, while old-old adults may face “othering” and increasing societal stigma [17, 18]. Despite stereotypes portraying older adults as digitally incompetent, research indicates that older adults (including old-old adults) can meaningfully engage with digital technologies when conditions allow [19, 20]. Empirical evidence shows differences between young-old and old-old adults were noted in various settings, such as psychological well-being [21], healthcare utilisation [22], and media representations [23]. However, digital inequality in various old age groups is yet to be determined. As shown in several studies of new media technologies, generation can be a predictor of technology use [24–28]. Differences in technology adoption and usage across age cohorts may be explained by social identity, worldviews, and life-course factors in a historical context [29–31]. In this context, it is hypothesized that digital inequalities may be present across young-old and old-old adults.

Our study departs from this theoretical understanding, focusing on the young-old and old-old age groups when examining digital inequality in Chinese older adults. While acknowledging the lack of consensus and potential problems associated with age groupings [32], our study does not seek to contribute to the academic discourse on defining old age. Instead, it aims to explore digital inequalities within these socially constructed and analytically feasible age cohorts from a social and cultural gerontological perspective. Our study’s results aim to potentially broaden the conventional understanding of digital inequalities, moving beyond the comparison solely between young and older adults. By examining two cohorts of advanced age, we seek to enhance the understanding of digital inequality and its underlying mechanisms within the older adult population.

## Materials and methods

### Study sample

Our study used data from the 2018 China Longitudinal Ageing Social Survey (CLASS), the first wave of this survey project to collect information on internet and technology use among older adults. Managed by the National Survey Research Centre at Renmin University of China, CLASS is a nationwide project that conducts baseline surveys every two years. The original survey was approved by Renmin University of China, and the author responsible for this study passed the ethics review conducted by Peking University, with the reference number 20221226025. Verbal informed consent was obtained from all individual participants included in the study, and the interviewer documented detailed information about the consent process.

### Instruments

**Digital inequality.** Our study employed a multidimensional approach to evaluate digital inequality among older adults. To assess *access to the Internet*, participants were asked about the availability of wired or wireless Internet in their area. Responses were coded as binary variables, with “yes” (= 1) indicating Internet access and “no” (= 0) indicating a lack thereof. The *frequency of Internet use* was determined by asking participants how often they used the Internet through various digital devices, with responses ranging from “never” to “every day”. Responses were coded on a scale of 0 to 4 (0 = “never”, 1 = “several times a year”, 2 = “at least once a month”, 3 = “at least once a week”, 4 = “everyday”), with higher scores indicating more frequent Internet use. For the *breadth of Internet use*, participants were asked about the purposes with a bunch of activities offered as options, including voice/video chat, text chat, shopping, reading news, browsing articles/information other than news, listening to music/radio and watching videos, playing games, taking transportation, managing health, investing such as stocks and funds, and learning and training. Each response was coded as 1 if selected, and 0 otherwise. A sum score was calculated (ranged 0 to 11) to determine the breadth of activities in which participants engaged online. To assess *digital skills*, a 5-point Likert scale was used (1 = “very unskilled”, 5 = “very skilled”), with participants asked to rate their proficiency with Internet-related devices, including smartphone, computers, and tablets. Responses were averaged to obtain a score, with higher scores indicating greater digital proficiency. Finally, participants were asked where they learned to use the Internet, with options including family members, friends and neighbours, community, and other organizations. Each response was coded as 1 if selected, and 0 otherwise. A sum score was computed (ranged 0 to 4) to reflect the availability of social support for learning how to use the Internet, with higher scores indicating greate*r availability of social support*.

#### Socioeconomic status

In this study, two socioeconomic status indicators were utilized: highest educational attainment and household income. *Highest level of education* was self-reported by participants at 5 levels: illiteracy, primary school, junior high school, high school, junior college, bachelor’s degree and above. *Household income* was assessed by asking respondents to indicate their average monthly household income over the past 12 months, measured in Chinese Yuan. The use of household income as a measure is justified by the fact that for 42% of older individuals, their family members are their primary source of income [33].

#### Covariates

Our study included several demographic and health factors as covariates, as previous research has established their association with digital access, usage, or skills [34–37]. Specifically, we controlled for age, gender, ethnicity, marital status, living arrangement, and geographic location of residence. Ethnicity was measured as Han or minority, marital status was categorized as married with a spouse or others, living arrangements were classified as living alone or with others, and self-reported location of residence was classified as urban, suburban, or rural. All classifications were reported by respondents. Moreover, our study considered the association of health and disability with digital inequalities, which has been identified by previous studies [4, 38]. Specifically, two indicators were used as covariates: self-reported health status and the need for health centre services. Self-reported health status was assessed using a 5-point Likert scale ranging from 1 (very unhealthy) to 5 (very healthy), while the need for health centre services was measured by asking respondents about their need for services such as home care, home doctor, rehabilitation training, rental of rehabilitation aids, free physical examination, establishment of health records, and health lectures. Responses were coded as 1 for “yes” and 0 for “no”, and a composite score was generated with a range of 0 to 7, where higher scores indicate a greater need for health services.

### Statistical analysis

The analysis of digital inequality among older adults was conducted on two age groups: young-old adults (60–74 years old) and old-old adults (75 years and older). A linear age measure was used in the non-stratified analyses to ensure the robustness of the findings. Chi-square tests, Kruskal-Wallis H tests, and hierarchical multiple regression were used to examine the relationship between socioeconomic status and digital use among older adults. For all analyses, a *p* < 0.05 was considered to be statistically significant. Control variables were included in Model 1, education and personal income in Model 2, and interaction terms of age group and socioeconomic status in Model 3 to assess any differences in the effect of socioeconomic status on digital inequality across age groups. The latter models had a better fit as indicated by the increased R square. SPSS 26.0 software was used for all analyses. Diagnostics to avoid collinearity (variance inflation factor <10.0) and interaction effect tests of independent variables (p > 0.05) were carried out. Normal distribution of residuals was proofed.

## Results

All completed responses were included in this study. The 2018 survey had 11,419 respondents, of which 69.55% were young-old adults (60–74 years old) and 30.45% were old-old adults (75+ years old). The ratio of young-old and old-old adults in our sample was 2.3:1, higher than the ratio recorded in the 2020 National Population Census (1.9:1). The number of female respondents (49.76%) was almost equal to the number of male respondents (50.24%), reflecting the gender distribution of China’s overall population. Most respondents had less than a middle school education (67%), with only 2% having a college education or higher. The majority of respondents were Han ethnicity (94.88%) and had no religious affiliation (93.18%). About 70% of respondents were married with a spouse, and 12% lived alone. Regarding residency, 45.83% of respondents lived in urban areas, 8.54% in suburban areas, and 45.63% in rural areas. This distribution of urban, suburban, and rural residency was slightly different from the 2020 National Population Census, where 33.6% of the population lived in urban areas, 20.0% in suburban areas, and 46.4% in rural areas.

The study found that 40.22% of older adults had access to wired or wireless internet, and 18.27% (N = 2086) were internet users (Table 1). Among Internet users, the majority are highly connected users who use the Internet every day (N = 1413).

**Table 1:** Descriptive results of Internet access and Frequency of Internet use.

	Older adults (N = 11,419)	Young-old adults (N = 7,942)	Old-old adults (N = 3,477)
	Frequency (Percentage)/ Mean (SD)	Frequency (Percentage)/ Mean (SD)	Frequency (Percentage)/ Mean (SD)
Internet access (Yes)	4,593 (40.22%)	3,453 (43.48%)	1,140 (32.79%)
**Frequency of Internet use**	0.65 (1.41)	0.86 (1.57)	0.16 (0.73)
Never	9,333 (81.73%)	6,038 (76.03%)	3,295 (94.77%)
Several times a year	89 (0.78%)	70 (0.88%)	19 (0.55%)
At least once a month	107 (0.94%)	86 (1.08%)	21 (0.60%)
At least once a week	477 (4.18%)	416 (5.24%)	61 (1.75%)
Every day	1,413 (12.37%)	1,332 (16.77%)	81 (2.33%)

As shown in Table 2, the main purposes of internet use were communication and information, with voice/video chat (85.47%), news reading (61.17%), and text chat (57.24%) being the most frequent. Older adults showed greater proficiency in using smartphones (M = 3.58, SD = 0.85) than personal computers (M = 2.19, SD = 1.16) or tablets (M = 1.99, SD = 1.06). The majority learned how to use the internet through family (74.30%) and friends/neighbours (45.35%), indicating a lack of community support. In terms of age differences, the old-old adults were more likely to use the internet for reading news than the young-old.

**Table 2:** Descriptive results of the breadth of Internet use, digital skills, and availability of social support.

	Older adults (N = 2,086)	Young-old adults (N = 1,904)	Old-old adults (N = 182)
	Frequency (Percentage)/ Mean (SD)	Frequency (Percentage)/ Mean (SD)	Frequency (Percentage)/ Mean (SD)
**Breadth of Internet use (Yes)**	3.29 (1.75)	3.35 (1.77)	2.63 (1.34)
Voice/video chat	1,783 (85.47%)	1,644 (86.34%)	139 (76.37%)
Text chat	1,194 (57.24%)	1,119 (58.77%)	75 (41.21%)
Shopping	273 (13.09%)	263 (13.81%)	10 (5.49%)
Reading news	1,276 (61.17%)	1,159 (60.87%)	117 (64.29%)
Browsing articles/information other than news	542 (25.98%)	520 (27.31%)	22 (12.09%)
Listening to music/radio and watching videos	992 (47.56%)	920 (48.32%)	72 (39.56%)
Playing games	423 (20.28%)	401 (21.06%)	22 (12.09%)
Taking transportation	170 (8.15%)	163 (8.56%)	7 (3.85%)
Managing health	85 (4.07%)	82 (4.31%)	3 (1.65%)
Investing such as stocks and funds	112 (5.37%)	102 (5.36%)	10 (5.49%)
Learning and training	8 (0.38%)	7 (0.37%)	1 (0.55%)
**Digital skills**	2.59 (0.85)	2.60 (0.85)	2.41 (0.81)
Smartphone	3.58 (0.85)	3.60 (0.84)	3.38 (0.90)
Very unskilled	39 (1.87%)	32 (1.68%)	7 (3.85%)
Unskilled	147 (7.05%)	131 (6.88%)	16 (8.79%)
Moderate	701 (33.60%)	627 (32.93%)	74 (40.66%)
Skilled	967 (46.36%)	897 (47.11%)	70 (38.46%)
Very skilled	232 (11.12%)	217 (11.40%)	15 (8.24%)
Computers	2.19 (1.16)	2.20 (1.15)	2.08 (1.18)
Very unskilled	784 (37.58%)	704 (36.98%)	80 (43.96%)
Unskilled	500 (23.97%)	460 (24.16%)	40 (21.98%)
Moderate	491 (23.54%)	453 (23.79%)	38 (20.88%)
Skilled	243 (11.65%)	227 (11.92%)	16 (8.79%)
Very skilled	68 (3.26%)	60 (3.15%)	8 (4.39%)
Tablets	1.99 (1.06)	2.02 (1.06)	1.77 (0.99)
Very unskilled	920 (44.10%)	819 (43.02%)	101 (55.49%)
Unskilled	486 (23.30%)	452 (23.74%)	34 (18.68%)
Moderate	479 (22.96%)	442 (23.21%)	37 (20.33%)
Skilled	175 (8.39%)	167 (8.77%)	8 (4.40%)
Very skilled	26 (1.25%)	24 (1.26%)	2 (1.10%)
**Availability of social support (Yes)**	1.22 (0.70)	1.23 (0.71)	1.12 (0.65)
Learn from family members (e.g., children)	1,550 (74.30%)	1,417 (74.42%)	133 (73.08%)
Learn from friends and neighbors	946 (45.35%)	882 (46.32%)	64 (35.14%)
Learn from community activities (e.g., public lectures, group learning, volunteer services)	44 (2.11%)	38 (2.00%)	6 (3.30%)
Participate in training activities organized by organizations other than communities	13 (0.62%)	12 (0.63%)	1 (0.55%)
Others	0 (0%)	0 (0%)	0 (0%)

To further understand the differences in digital inequality between young-old adults (aged 60–74) and old-old adults (aged 75 and older), detailed comparisons were conducted. The results showed that the old-old adults generally experienced a disadvantage in all aspects of digital inequality compared to the young-old adults. Specifically, the old-old adults (32.79%) had access to the Internet compared with younger counterparts (43.48%) (*X*^*2*^ = 114.954, *p* < 0.001). In terms of both frequency (*X*^*2*^ = 584.520, *p* < 0.001) and breadth (*X*^*2*^ = 26.700, *p* < 0.001) of Internet use, old-old adults (M_frenquency_ = 0.16, SD_frenquency_ = 0.73; M_breadth_ = 2.63, SD_breadth_ = 1.34) are disadvantaged than young-old adults (M_frenquency_ = 0.86, SD_frenquency_ = 1.57; M_breadth_ = 3.35, SD_breadth_ = 1.77). Old-old people were disadvantaged in using smartphones (M = 3.38, SD = 0.90) and tablets (M = 1.77, SD = 0.99) and in receiving support from friends and neighbours (35.14%) than the young-old. These findings were supported by Kruskal-Wallis H tests and Chi-square tests (see Table 3).

**Table 3:** Results of Kruskal-Wallis H tests and Chi-square tests between young-old adults and old-old adults.

	*X* ^*2*^	df	N	*p*
Internet access	114.954	1	11419	< 0.001
Frequency of Internet use	584.520	1	11419	< 0.001
**Breadth of Internet use**	26.700	1	2086	< 0.001
Voice/video chat	13.302	1	2086	< 0.001
Text chat	20.934	1	2086	< 0.001
Shopping	10.106	1	2086	0.001
Reading news	0.815	1	2086	0.367
Browsing articles/information other than news	20.017	1	2086	< 0.001
Listening to music/radio and watching videos	5.110	1	2086	0.024
Playing games	8.274	1	2086	0.004
Taking transportation	4.933	1	2086	0.026
Managing health	3.003	1	2086	0.083
Investing such as stocks and funds	0.006	1	2086	0.937
Learning and training	0.144	1	2086	0.705
**Digital skills**	8.092	1	2086	0.004
Smartphone	9.937	1	2086	0.002
Computers	2.700	1	2086	0.100
Tablets	9.888	1	2086	0.002
**Availability of social support**	5.201	1	2086	0.023
Learn from family members (e.g., children)	0.157	1	2086	0.691
Learn from friends and neighbours	8.346	1	2086	0.004
Learn from community activities (e.g., public lectures, group learning, volunteer services)	1.362	1	2086	0.243
Participate in training activities organized by organizations other than communities	0.018	1	2086	0.895

Our study analysed whether different socioeconomic factors affect digital inequality in two age groups: young-old adults and old-old adults. The impact of socioeconomic factors was found to differ between two groups (see Table 4 for details). For example, the highest educational attainment was positively associated with all dimensions of Internet use in young-old adults, but only had a partially positive association with some dimensions in old-old adults (i.e., Internet access, frequency of Internet use and availability of social support). Additionally, higher household income was linked to greater Internet access and use in young-old adults, while to greater Internet access, breadth, and digital skills in old-old adults. However, the impact of income was relatively small.

**Table 4:** Main results of regression between young-old adults and old-old adults.

	Internet access			
	Young-old adults		Old-old adults	
	B [95% Confidence Interval]	β	B [95% Confidence Interval]	β
*Covariates*				
Age	-0.016 [-0.019, -0.014]	-0.123	-0.0004 [-0.004, 0.003]	-0.004
Gender (male)	-0.036 [-0.057, -0.016]	-0.036	-0.008 [-0.040, 0.024]	-0.008
Ethnicity (Han)	-0.031 [-0.078, 0.016]	-0.014	-0.124 [-0.190, -0.058]	-0.061
Marital status (married with spouse)	-0.056 [-0.084, -0.027]	-0.047	-0.086 [-0.121, -0.051]	-0.092
Living arrangement (alone)	-0.187 [-0.226, -0.147]	-0.110	-0.200 [-0.242, -0.158]	-0.165
Residence (suburban)	-0.095 [-0.132, -0.057]	-0.054	-0.132 [-0.189, -0.074]	-0.076
Residence (rural)	-0.189 [-0.212, -0.167]	-0.190	-0.157 [-0.191, -0.123]	-0.166
Self-reported health status	0.023 [0.011, 0.035]	0.041	0.022 [0.006, 0.038]	0.043
Needs for health center services	0.017 [0.012, 0.022]	0.066	0.010 [0.002, 0.018]	0.042
*Socioeconomic status*				
Education (primary school)	0.095 [0.068, 0.122]	0.095	0.054 [0.019, 0.089]	0.056
Education (junior high school)	0.220 [0.189, 0.251]	0.197	0.085 [0.036, 0.134]	0.064
Education (high school)	0.292 [0.248, 0.335]	0.163	0.137 [0.067, 0.206]	0.068
Education (junior college)	0.413 [0.332, 0.494]	0.108	0.310 [0.199, 0.420]	0.093
Education (bachelor’s degree and above)	0.461 [0.260, 0.661]	0.047	0.248 [0.012, 0.485]	0.034
Monthly household income	0.0006 [0.0004, 0.0008]	0.065	0.0009 [0.0005, 0.001]	0.084
	**Frequency of Internet Use**			
	**Young-old adults**		**Old-old adults**	
	B [95% Confidence Interval]	β	B [95% Confidence Interval]	β
*Covariates*				
Age	-0.079 [-0.087, -0.070]	-0.186	-0.011 [-0.016, -0.005]	-0.069
Gender (male)	-0.040 [-0.103, 0.023]	-0.013	0.025 [-0.026, 0.076]	0.017
Ethnicity (Han)	0.055 [-0.088, 0.198]	0.008	0.117 [0.012, 0.221]	0.037
Marital status (married with spouse)	0.060 [-0.027, 0.147]	0.016	0.003 [-0.053, 0.058]	0.002
Living arrangement (alone)	-0.122 [-0.242, -0.002]	-0.023	-0.056 [-0.123, 0.011]	-0.030
Residence (suburban)	-0.351 [-0.465, -0.237]	-0.063	-0.089 [-0.181, 0.003]	-0.033
Residence (rural)	-0.476 [-0.546, -0.406]	-0.151	-0.045 [-0.100, 0.009]	-0.031
Self-reported health status	0.044 [0.009, 0.080]	0.025	0.004 [-0.022, 0.030]	0.005
Needs for health center services	0.046 [0.030, 0.062]	0.057	0.005 [-0.007, 0.018]	0.014
*Socioeconomic status*				
Education (primary school)	0.187 [0.105, 0.270]	0.059	0.056 [0.001, 0.111]	0.037
Education (junior high school)	0.838 [0.743, 0.932]	0.236	0.244 [0.166, 0.322]	0.118
Education (high school)	1.355 [1.223, 1.487]	0.238	0.270 [0.159, 0.381]	0.086
Education (junior college)	1.718 [1.468, 1.967]	0.141	0.535 [0.359, 0.711]	0.103
Education (bachelor’s degree and above)	2.231 [1.615, 2.847]	0.071	1.321 [0.944, 1.698]	0.115
Monthly household income	0.001 [0.0004, 0.002]	0.034	0.0002 [-0.0003, 0.0008]	0.013
	**Breadth of Internet Use**			
	**Young-old adults**		**Old-old adults**	
	B [95% Confidence Interval]	β	B [95% Confidence Interval]	β
*Covariates*				
Age	-0.089 [-0.112, -0.067]	-0.175	-0.020 [-0.064, 0.025]	-0.067
Gender (male)	0.309 [0.153, 0.465]	0.087	-0.018 [-0.447, 0.411]	-0.007
Ethnicity (Han)	0.425 [-0.016, 0.866]	0.042	1.736 [0.131, 3.340]	0.165
Marital status (married with spouse)	0.019 [-0.228, 0.267]	0.004	0.049 [-0.427, 0.525]	0.018
Living arrangement (alone)	0.299 [-0.068, 0.666]	0.040	0.072 [-0.605, 0.748]	0.017
Residence (suburban)	-0.100 [-0.395, 0.194]	-0.015	0.324 [-0.574, 1.223]	0.055
Residence (rural)	-0.509 [-0.701, -0.316]	-0.123	0.406 [-0.093, 0.906]	0.141
Self-reported health status	0.031 [-0.060, 0.123]	0.015	-0.047 [-0.274, 0.180]	-0.033
Needs for health center services	0.061 [0.025, 0.097]	0.075	0.035 [-0.063, 0.133]	0.054
*Socioeconomic status*				
Education (primary school)	0.922 [0.583, 1.260]	0.227	0.168 [-0.501, 0.838]	0.060
Education (junior high school)	0.853 [0.519, 1.187]	0.239	-0.035 [-0.739, 0.669]	-0.012
Education (high school)	1.017 [0.651, 1.384]	0.225	0.081 [-0.782, 0.943]	0.019
Education (junior college)	1.493 [1.019, 1.967]	0.182	0.198 [-0.788, 1.184]	0.038
Education (bachelor’s degree and above)	2.149 [1.233, 3.064]	0.107	0.338 [-0.973, 1.648]	0.045
Monthly household income	0.0006 [-0.001, 0.002]	0.017	0.002 [0.0006, 0.003]	0.249
	**Breadth of Internet Use**			
	**Young-old adults**		**Old-old adults**	
	B [95% Confidence Interval]	β	B [95% Confidence Interval]	β
*Covariates*				
Age	-0.089 [-0.112, -0.067]	-0.175	-0.020 [-0.064, 0.025]	-0.067
Gender (male)	0.309 [0.153, 0.465]	0.087	-0.018 [-0.447, 0.411]	-0.007
Ethnicity (Han)	0.425 [-0.016, 0.866]	0.042	1.736 [0.131, 3.340]	0.165
Marital status (married with spouse)	0.019 [-0.228, 0.267]	0.004	0.049 [-0.427, 0.525]	0.018
Living arrangement (alone)	0.299 [-0.068, 0.666]	0.040	0.072 [-0.605, 0.748]	0.017
Residence (suburban)	-0.100 [-0.395, 0.194]	-0.015	0.324 [-0.574, 1.223]	0.055
Residence (rural)	-0.509 [-0.701, -0.316]	-0.123	0.406 [-0.093, 0.906]	0.141
Self-reported health status	0.031 [-0.060, 0.123]	0.015	-0.047 [-0.274, 0.180]	-0.033
Needs for health center services	0.061 [0.025, 0.097]	0.075	0.035 [-0.063, 0.133]	0.054
*Socioeconomic status*				
Education (primary school)	0.922 [0.583, 1.260]	0.227	0.168 [-0.501, 0.838]	0.060
Education (junior high school)	0.853 [0.519, 1.187]	0.239	-0.035 [-0.739, 0.669]	-0.012
Education (high school)	1.017 [0.651, 1.384]	0.225	0.081 [-0.782, 0.943]	0.019
Education (junior college)	1.493 [1.019, 1.967]	0.182	0.198 [-0.788, 1.184]	0.038
Education (bachelor’s degree and above)	2.149 [1.233, 3.064]	0.107	0.338 [-0.973, 1.648]	0.045
Monthly household income	0.0006 [-0.001, 0.002]	0.017	0.002 [0.0006, 0.003]	0.249
	**Availability of social support**			
	**Young-old adults**		**Old-old adults**	
	B [95% Confidence Interval]	β	B [95% Confidence Interval]	β
*Covariates*				
Age	0.003 [-0.006, 0.012]	0.015	0.001 [-0.021, 0.022]	0.004
Gender (male)	-0.057 [-0.121, 0.007]	-0.040	0.019 [-0.187, 0.225]	0.014
Ethnicity (Han)	-0.021 [-0.202, 0.161]	-0.005	0.547 [-0.223, 1.316]	0.107
Marital status (married with spouse)	0.005 [-0.097, 0.107]	0.002	-0.153 [-0.381, 0.075]	-0.114
Living arrangement (alone)	-0.079 [-0.230, 0.072]	-0.027	0.064 [-0.260, 0.388]	0.031
Residence (suburban)	-0.010 [-0.131, 0.112]	-0.004	0.226 [-0.205, 0.657]	0.079
Residence (rural)	-0.073 [-0.152, 0.006]	-0.044	-0.095 [-0.335, 0.145]	-0.067
Self-reported health status	-0.031 [-0.069, 0.007]	-0.038	0.056 [-0.053, 0.165]	0.080
Needs for health center services	0.018 [0.003, 0.033]	0.055	0.006 [-0.041, 0.053]	0.020
*Socioeconomic status*				
Education (primary school)	0.345 [0.206, 0.484]	0.213	0.410 [0.089, 0.731]	0.299
Education (junior high school)	0.429 [0.291, 0.566]	0.301	0.466 [0.129, 0.804]	0.329
Education (high school)	0.485 [0.334, 0.636]	0.269	0.269 [-0.144, 0.683]	0.132
Education (junior college)	0.262 [0.067, 0.457]	0.080	0.703 [0.230, 1.176]	0.278
Education (bachelor’s degree and above)	0.549 [0.172, 0.925]	0.069	0.656 [0.027, 1.284]	0.180
Monthly household income	-0.0004 [-0.001, 0.0002]	-0.026	-0.0008 [-0.006, 0.004]	-0.025

A hierarchical multiple regression analysis of the entire sample was performed, and it was found that age had a moderating effect on the relationship between socioeconomic status and outcome variables other than availability of social support (see supplementary material for details). In other words, the association of socioeconomic factors with Internet use varied depending on age. Older adults who lived alone were found to have lower level of Internet access [β = -0.131, B = -0.196, 95% CI: (-0.225, -0.167)] and lower frequency of Internet use [β = -0.025, B = -0.108, 95% CI: (-0.186, -0.029)]. Compared with older adults living in urban areas, those in rural areas were disadvantaged in all dimensions of Internet use.

## Discussion

Our study aimed to investigate digital inequalities among older adults using a nationally representative sample in China, with a focus on young-old and old-old adults. Our results show that old-old adults faced more digital disadvantages compared to young-old adults, which is consistent with previous studies that found a digital divide in old age cohorts [4]. This gap might be explained by a lack of need to use the Internet or a lack of income and social support for Internet use in very old age [39]. As evidenced in our study, digital inequality manifests in various aspects of digital access and use. It was also found to be associated with socioeconomic factors. This finding resonates with previous studies [8, 10, 40], which suggest that certain subpopulations of older adults, such as those with low socioeconomic status, older women, those in long-term care facilities, and the old-old, are particularly susceptible to digital disadvantages.

Our study specifically found that socioeconomic status had an association with various dimensions of digital inequality among older adults, and the association differed by age cohort. Among young-old adults in our study, higher education and economic status were associated with more frequent Internet use, better digital skills, and greater social support. This suggests that those with lower socioeconomic status in this group have a disadvantage in using digital technology and accessing social support. This finding has implications for encouraging targeted interventions to address digital inequalities in the older adult population. For young-old adults, interventions could focus on providing digital literacy training programs that enhance their digital skills and promote the use of the Internet for social connections and information seeking. Such programs could be offered through community centres, libraries, and senior centres, especially for those with lower socioeconomic status [41]. As found in our study, among old-old adults, lower economic status was linked to lower levels of Internet access and available social support. This suggests that for old-old adults, interventions aimed at increasing their social resources could be more essential to tackling their digital disadvantages. Overall, interventions should be tailored to the specific and heterogeneous needs and conditions of older adult subpopulations, thus potentially leading to reduced digital inequalities.

Tackling digital inequality is a critical issue on ageing policy agendas. The United Nations recognizes Internet access as a human right and has called for advancing digital equity for all ages, emphasizing the significance of older adults having equitable access and meaningful engagement in the digital world [42, 43]. Digital inclusion is emerging as a critical factor in promoting healthy ageing from a public health perspective. Technology is seen as a critical component of ageing environments and offers opportunities to enhance digital opportunities for older adults. To address digital inequality among older adults in China, policymakers should consider the following policy recommendations: firstly, prioritize initiatives that promote affordable access to digital technology, especially for the most vulnerable subpopulations such as the old-old and those with low socioeconomic status. Secondly, the government should invest in digital literacy programs to increase the digital skills of older adults. Lastly, the government should encourage the development of social support networks that cater to the specific needs of older adults.

Our study has some limitations that warrant consideration. First, the sample size for the old-old adults is limited, which may result in random errors. Second, our study relies on cross-sectional data to examine the relationship between socioeconomic status and digital experience, and future research using longitudinal data can provide more insights into the cumulative effects of socioeconomic disadvantages on digital inequalities in old age. Finally, qualitative research can further shed light on cultural and generational differences in Internet and technology adoption and use by young-old and old-old adults. These gaps present opportunities for future research.

## Supporting information

S1 Table Results of hierarchical multiple regression for sampled older adults. (DOCX)

S2 Table Results of hierarchical multiple regression for young-old adults. (DOCX)

S3 Table Results of hierarchical multiple regression for old-old adults. (DOCX)
