# Supplementary material for: Socioeconomic differences in digital inequality among Chinese older adults: Results from a nationally representative sample
Source: PLoS One. 2024 Apr 2;19(4):e0300433. doi: 10.1371/journal.pone.0300433 (PMC10986962; doi:10.1371/journal.pone.0300433)
Supplement: S1 Table — (DOCX) [file pone.0300433.s001.docx]

**S1 Table. Results of** **hierarchical multiple regression for sampled older adults.**

|  | **Internet access** | | | | | | | | |
| --- | --- | --- | --- | --- | --- | --- | --- | --- | --- |
|  | Model 1a | | | Model 2a | | | Model 3a | | |
|  | B [95% Confidence Interval] | β | *p* | B [95% Confidence Interval] | β | *p* | B [95% Confidence Interval] | β | *p* |
| *Covariates* |  |  |  |  |  |  |  |  |  |
| Age | -0.009 [-0.010, -0.008] | -0.137 | < 0.001 | -0.007 [-0.008, -0.006] | -0.108 | < 0.001 | -0.008 [-0.009, -0.006] | -0.116 | < 0.001 |
| Gender (male) | -0.006 [-0.024, 0.011] | -0.006 | 0.485 | -0.031 [-0.048, -0.013] | -0.031 | 0.001 | -0.030 [-0.047, -0.012] | -0.030 | 0.001 |
| Ethnicity (Han) | -0.056 [-0.095, -0.017] | -0.025 | 0.005 | -0.060 [-0.099, -0.022] | -0.027 | 0.002 | -0.060 [-0.099, -0.022] | -0.027 | 0.002 |
| Marital status (married with spouse) | -0.059 [-0.081, -0.037] | -0.055 | < 0.001 | -0.076 [-0.098, -0.053] | -0.071 | < 0.001 | -0.072 [-0.094, -0.050] | -0.067 | < 0.001 |
| Living arrangement (alone) | -0.205 [-0.234, -0.175] | -0.137 | < 0.001 | -0.198 [-0.227, -0.169] | -0.132 | < 0.001 | -0.196 [-0.225, -0.167] | -0.131 | < 0.001 |
| Residence (suburban) | -0.149 [-0.181, -0.117] | -0.085 | < 0.001 | -0.106 [-0.137, -0.075] | -0.06 | < 0.001 | -0.105 [-0.137, -0.074] | -0.060 | < 0.001 |
| Residence (rural) | -0.253 [-0.271, -0.235] | -0.257 | < 0.001 | -0.181 [-0.200, -0.162] | -0.184 | < 0.001 | -0.180 [-0.199, -0.161] | -0.183 | < 0.001 |
| Self-reported health status | 0.033 [0.023, 0.043] | 0.060 | < 0.001 | 0.025 [0.015, 0.034] | 0.045 | < 0.001 | 0.024 [0.015, 0.034] | 0.044 | < 0.001 |
| Needs for health center services | 0.017 [0.013, 0.022] | 0.068 | < 0.001 | 0.015 [0.011, 0.019] | 0.059 | < 0.001 | 0.015 [0.011, 0.019] | 0.059 | < 0.001 |
| *Socioeconomic status* |  |  |  |  |  |  |  |  |  |
| Education (primary school) |  |  |  | 0.071 [0.050, 0.093] | 0.071 | < 0.001 | 0.080 [0.059, 0.102] | 0.080 | < 0.001 |
| Education (junior high school) |  |  |  | 0.184 [0.158, 0.210] | 0.158 | < 0.001 | 0.181 [0.155, 0.207] | 0.156 | < 0.001 |
| Education (high school) |  |  |  | 0.251 [0.215, 0.288] | 0.135 | < 0.001 | 0.250 [0.214, 0.287] | 0.135 | < 0.001 |
| Education (junior college) |  |  |  | 0.381 [0.316, 0.447] | 0.103 | < 0.001 | 0.391 [0.325, 0.457] | 0.106 | < 0.001 |
| Education (bachelor’s degree and above) |  |  |  | 0.378 [0.224, 0.532] | 0.042 | < 0.001 | 0.403 [0.246, 0.560] | 0.045 | < 0.001 |
| Monthly household income |  |  |  | 0.0001 [0.000, 0.0005] | 0.069 | < 0.001 | 0.0007 [0.0005, 0.0009] | 0.071 | < 0.001 |
| *Interaction terms* |  |  |  |  |  |  |  |  |  |
| Age group × Education (primary school) |  |  |  |  |  |  | -0.014 [-0.024, -0.004] | -0.028 | 0.005 |
| Age group × Education (junior high school) |  |  |  |  |  |  | -0.035 [-0.045, -0.024] | -0.065 | < 0.001 |
| Age group × Education (high school) |  |  |  |  |  |  | -0.025 [-0.035, -0.016] | -0.048 | < 0.001 |
| Age group × Education (junior college) |  |  |  |  |  |  | -0.009 [-0.018, -0.001] | -0.020 | 0.026 |
| Age group × Education (bachelor’s degree and above) |  |  |  |  |  |  | -0.006 [-0.014, 0.001] | -0.014 | 0.112 |
| Age group × Monthly household income |  |  |  |  |  |  | 0.006 [-0.003, 0.016] | 0.012 | 0.176 |
| R2 | 0.110 | | | 0.143 | | | 0.148 | | |
| Adjusted R2 | 0.109 | | | 0.142 | | | 0.146 | | |
| F | 156.15 | | | 127.186 | | | 94.006 | | |
| DF | 9, 11409 | | | 15, 11403 | | | 21, 11397 | | |

**S1 Table Continued.**

|  | **Frequency of Internet Use** | | | | | | | | |
| --- | --- | --- | --- | --- | --- | --- | --- | --- | --- |
|  | Model 1b | | | Model 2b | | | Model 3b | | |
|  | B [95% Confidence Interval] | β | *p* | B [95% Confidence Interval] | β | *p* | B [95% Confidence Interval] | β | *p* |
| *Covariates* |  |  |  |  |  |  |  |  |  |
| Age | -0.054 [-0.057, -0.050] | -0.280 | < 0.001 | -0.046 [-0.049, -0.042] | -0.24 | < 0.001 | -0.048 [-0.052, -0.045] | -0.252 | < 0.001 |
| Gender (male) | 0.052 [0.003, 0.101] | 0.019 | 0.037 | -0.032 [-0.080, 0.016] | -0.011 | 0.19 | -0.025 [-0.072, 0.023] | -0.009 | 0.308 |
| Ethnicity (Han) | 0.077 [-0.033, 0.186] | 0.012 | 0.170 | 0.074 [-0.032, 0.179] | 0.012 | 0.17 | 0.077 [-0.027, 0.181] | 0.012 | 0.149 |
| Marital status (married with spouse) | 0.058 [-0.005, 0.121] | 0.019 | 0.070 | 0.001 [-0.006, 0.062] | 0.0003 | 0.975 | 0.024 [-0.036, 0.084] | 0.008 | 0.431 |
| Living arrangement (alone) | -0.142 [-0.225, -0.059] | -0.033 | 0.001 | -0.120 [-0.199, -0.040] | -0.028 | 0.003 | -0.108 [-0.186, -0.029] | -0.025 | 0.007 |
| Residence (suburban) | -0.437 [-0.526, -0.348] | -0.087 | < 0.001 | -0.275 [-0.362, -0.188] | -0.055 | < 0.001 | -0.275 [-0.360, -0.189] | -0.055 | < 0.001 |
| Residence (rural) | -0.614 [-0.665, -0.564] | -0.217 | < 0.001 | -0.351 [-0.404, -0.299] | -0.124 | < 0.001 | -0.350 [-0.402, -0.298] | -0.124 | < 0.001 |
| Self-reported health status | 0.077 [0.050, 0.104] | 0.049 | < 0.001 | 0.044 [0.018, 0.071] | 0.028 | 0.001 | 0.039 [0.013, 0.065] | 0.025 | 0.003 |
| Needs for health center services | 0.041 [0.029, 0.054] | 0.057 | < 0.001 | 0.033 [0.021, 0.045] | 0.046 | < 0.001 | 0.034 [0.022, 0.046] | 0.046 | < 0.001 |
| *Socioeconomic status* |  |  |  |  |  |  |  |  |  |
| Education (primary school) |  |  |  | 0.079 [0.020, 0.138] | 0.028 | 0.008 | 0.134 [0.075, 0.193] | 0.047 | < 0.001 |
| Education (junior high school) |  |  |  | 0.670 [0.599, 0.742] | 0.201 | < 0.001 | 0.669 [0.597, 0.740] | 0.200 | < 0.001 |
| Education (high school) |  |  |  | 1.07 [0.970, 0.171] | 0.201 | < 0.001 | 1.043 [0.943, 1.144] | 0.196 | < 0.001 |
| Education (junior college) |  |  |  | 1.291 [1.110, 1.473] | 0.122 | < 0.001 | 1.389 [1.209, 1.569] | 0.131 | < 0.001 |
| Education (bachelor’s degree and above) |  |  |  | 1.828 [1.403, 2.253] | 0.071 | < 0.001 | 1.977 [1.548, 2.407] | 0.077 | < 0.001 |
| Monthly household income |  |  |  | 0.0008 [0.0003, 0.001] | 0.029 | 0.001 | 0.0007 [0.0003, 0.001] | 0.026 | 0.002 |
| *Interaction terms* |  |  |  |  |  |  |  |  |  |
| Age group × Education (primary school) |  |  |  |  |  |  | -0.059 [-0.086, -0.032] | -0.042 | < 0.001 |
| Age group × Education (junior high school) |  |  |  |  |  |  | -0.168 [-0.197, -0.139] | -0.109 | < 0.001 |
| Age group × Education (high school) |  |  |  |  |  |  | -0.175 [-0.201, -0.149] | -0.116 | < 0.001 |
| Age group × Education (junior college) |  |  |  |  |  |  | -0.095 [-0.118, -0.073] | -0.070 | < 0.001 |
| Age group × Education (bachelor’s degree and above) |  |  |  |  |  |  | -0.031 [-0.053, -0.010] | -0.024 | 0.004 |
| Age group × Monthly household income |  |  |  |  |  |  | -0.029 [-0.054, -0.004] | -0.019 | 0.024 |
| R2 | 0.145 | | | 0.208 | | | 0.230 | | |
| Adjusted R2 | 0.145 | | | 0.207 | | | 0.228 | | |
| F | 215.454 | | | 199.971 | | | 161.964 | | |
| DF | 9, 11409 | | | 15, 11403 | | | 21, 11397 | | |

**S1 Table Continued.**

|  | **Breadth of Internet Use** | | | | | | | | |
| --- | --- | --- | --- | --- | --- | --- | --- | --- | --- |
|  | Model 1c | | | Model 2c | | | Model 3c | | |
|  | B [95% Confidence Interval] | β | *p* | B [95% Confidence Interval] | β | *p* | B [95% Confidence Interval] | β | *p* |
| *Covariates* |  |  |  |  |  |  |  |  |  |
| Age | -0.065 [-0.079, -0.050] | -0.196 | < 0.001 | -0.063 [-0.077, -0.049] | -0.191 | < 0.001 | -0.061 [-0.076, -0.045] | -0.183 | < 0.001 |
| Gender (male) | 0.309 [0.160, 0.457] | 0.088 | < 0.001 | 0.277 [0.129, 0.425] | 0.079 | < 0.001 | 0.275 [0.127, 0.422] | 0.078 | < 0.001 |
| Ethnicity (Han) | 0.375 [-0.053, 0.803] | 0.036 | 0.086 | 0.454 [0.028, 0.880] | 0.044 | 0.037 | 0.452 [0.027, 0.877] | 0.044 | 0.037 |
| Marital status (married with spouse) | -0.004 [-0.229, 0.221] | -0.001 | 0.973 | -0.034 [-0.258, 0.189] | -0.007 | 0.762 | -0.032 [-0.255, 0.191] | -0.007 | 0.780 |
| Living arrangement (alone) | 0.239 [-0.094, 0.571] | 0.034 | 0.160 | 0.284 [-0.047, 0.615] | 0.040 | 0.093 | 0.261 [-0.070, 0.592] | 0.037 | 0.123 |
| Residence (suburban) | -0.133 [-0.416, 0.151] | -0.020 | 0.359 | -0.069 [-0.351, 0.213] | -0.010 | 0.633 | -0.069 [-0.350, 0.213] | -0.010 | 0.633 |
| Residence (rural) | -0.590 [-0.761, -0.420] | -0.146 | < 0.001 | -0.411 [-0.592, -0.230] | -0.102 | < 0.001 | -0.404 [-0.585, -0.223] | -0.100 | < 0.001 |
| Self-reported health status | 0.067 [-0.019, 0.152] | 0.033 | 0.125 | 0.036 [-0.050, 0.121] | 0.018 | 0.413 | 0.033 [-0.052, 0.119] | 0.017 | 0.444 |
| Needs for health center services | 0.057 [0.023, 0.091] | 0.071 | 0.001 | 0.055 [0.022, 0.089] | 0.068 | 0.001 | 0.056 [0.022, 0.090] | 0.069 | 0.001 |
| *Socioeconomic status* |  |  |  |  |  |  |  |  |  |
| Education (primary school) |  |  |  | 0.782 [0.473, 1.091] | 0.197 | < 0.001 | 0.657 [0.321, 0.993] | 0.165 | < 0.001 |
| Education (junior high school) |  |  |  | 0.718 [0.411, 1.025] | 0.202 | < 0.001 | 0.507 [0.163, 0.850] | 0.143 | 0.004 |
| Education (high school) |  |  |  | 0.915 [0.576, 1.254] | 0.202 | < 0.001 | 0.684 [0.283, 1.085] | 0.151 | 0.001 |
| Education (junior college) |  |  |  | 1.336 [0.900, 1.772] | 0.168 | < 0.001 | 1.047 [0.563, 1.531] | 0.131 | < 0.001 |
| Education (bachelor’s degree and above) |  |  |  | 1.675 [0.898, 2.451] | 0.095 | < 0.001 | 1.444 [0.659, 2.229] | 0.082 | < 0.001 |
| Monthly household income |  |  |  | 0.0009 [-0.0007, 0.003] | 0.023 | 0.286 | 0.005 [0.002, 0.010] | 0.140 | 0.007 |
| *Interaction terms* |  |  |  |  |  |  |  |  |  |
| Age group × Education (primary school) |  |  |  |  |  |  | -0.188 [-0.378, 0.002] | -0.077 | 0.052 |
| Age group × Education (junior high school) |  |  |  |  |  |  | -0.227 [-0.393, -0.062] | -0.117 | 0.007 |
| Age group × Education (high school) |  |  |  |  |  |  | -0.152 [-0.279, -0.025] | -0.095 | 0.019 |
| Age group × Education (junior college) |  |  |  |  |  |  | -0.099 [-0.173, -0.025] | -0.077 | 0.009 |
| Age group × Education (bachelor’s degree and above) |  |  |  |  |  |  | -0.059 [-0.102, -0.015] | -0.060 | 0.008 |
| Age group × Monthly household income |  |  |  |  |  |  | 0.353 [0.074, 0.631] | 0.130 | 0.013 |
| R2 | 0.075 | | | 0.097 | | | 0.103 | | |
| Adjusted R2 | 0.071 | | | 0.090 | | | 0.094 | | |
| F | 18.619 | | | 14.76 | | | 11.281 | | |
| DF | 9, 2076 | | | 15, 2070 | | | 21, 2064 | | |

**S1 Table Continued.**

|  | **Digital skills** | | | | | | | | |
| --- | --- | --- | --- | --- | --- | --- | --- | --- | --- |
|  | Model 1d | | | Model 2d | | | Model 3d | | |
|  | B [95% Confidence Interval] | β | *p* | B [95% Confidence Interval] | β | *p* | B [95% Confidence Interval] | β | *p* |
| *Covariates* |  |  |  |  |  |  |  |  |  |
| Age | -0.015 [-0.022, -0.008] | -0.094 | < 0.001 | -0.012 [-0.019, -0.005] | -0.075 | 0.001 | -0.012 [-0.019, -0.004] | -0.072 | 0.003 |
| Gender (male) | 0.007 [-0.066, 0.080] | 0.004 | 0.848 | -0.012 [-0.084, 0.060] | -0.007 | 0.749 | -0.014 [-0.086, 0.058] | -0.008 | 0.697 |
| Ethnicity (Han) | -0.026 [-0.235, 0.184] | -0.005 | 0.808 | -0.023 [-0.231, 0.185] | -0.005 | 0.826 | -0.018 [-0.225, 0.189] | -0.004 | 0.866 |
| Marital status (married with spouse) | -0.066 [-0.176, 0.045] | -0.029 | 0.242 | -0.081 [-0.190, 0.028] | -0.036 | 0.145 | -0.071 [-0.180, 0.038] | -0.032 | 0.199 |
| Living arrangement (alone) | -0.133 [-0.296, 0.030] | -0.039 | 0.110 | -0.123 [-0.285, 0.039] | -0.036 | 0.136 | -0.127 [-0.289, 0.034] | -0.037 | 0.122 |
| Residence (suburban) | -0.103 [-0.242, 0.036] | -0.031 | 0.148 | -0.058 [-0.196, 0.080] | -0.018 | 0.410 | -0.060 [-0.197, 0.077] | -0.018 | 0.390 |
| Residence (rural) | -0.324 [-0.408, -0.240] | -0.165 | < 0.001 | -0.235 [-0.323, -0.147] | -0.120 | < 0.001 | -0.231 [-0.319, -0.142] | -0.118 | < 0.001 |
| Self-reported health status | 0.065 [0.024, 0.107] | 0.067 | 0.002 | 0.040 [-0.001, 0.082] | 0.041 | 0.058 | 0.046 [0.004, 0.087] | 0.047 | 0.032 |
| Needs for health center services | 0.034 [0.017, 0.050] | 0.085 | < 0.001 | 0.029 [0.012, 0.045] | 0.073 | 0.001 | 0.030 [0.013, 0.046] | 0.076 | < 0.001 |
| *Socioeconomic status* |  |  |  |  |  |  |  |  |  |
| Education (primary school) |  |  |  | -0.136 [-0.287, 0.015] | -0.071 | 0.077 | -0.092 [-0.256, 0.071] | -0.048 | 0.269 |
| Education (junior high school) |  |  |  | 0.111 [-0.039, 0.261] | 0.065 | 0.147 | 0.040 [-0.127, 0.207] | 0.023 | 0.641 |
| Education (high school) |  |  |  | 0.221 [0.056, 0.387] | 0.101 | 0.009 | 0.106 [-0.089, 0.301] | 0.048 | 0.287 |
| Education (junior college) |  |  |  | 0.349 [0.136, 0.562] | 0.090 | 0.001 | 0.187 [-0.049, 0.422] | 0.048 | 0.121 |
| Education (bachelor’s degree and above) |  |  |  | 0.003 [-0.377, 0.382] | 0.0003 | 0.989 | -0.072 [-0.454, 0.310] | -0.008 | 0.712 |
| Monthly household income |  |  |  | 0.0007 [-0.0001, 0.002] | 0.037 | 0.089 | 0.004 [0.002, 0.006] | 0.190 | < 0.001 |
| *Interaction terms* |  |  |  |  |  |  |  |  |  |
| Age group × Education (primary school) |  |  |  |  |  |  | 0.054 [-0.038, 0.146] | 0.046 | 0.252 |
| Age group × Education (junior high school) |  |  |  |  |  |  | -0.055 [-0.135, 0.025] | -0.059 | 0.180 |
| Age group × Education (high school) |  |  |  |  |  |  | -0.055 [-0.116, 0.007] | -0.070 | 0.082 |
| Age group × Education (junior college) |  |  |  |  |  |  | -0.048 [-0.084, -0.012] | -0.076 | 0.010 |
| Age group × Education (bachelor’s degree and above) |  |  |  |  |  |  | -0.017 [-0.038, 0.004] | -0.036 | 0.114 |
| Age group × Monthly household income |  |  |  |  |  |  | 0.226 [0.091, 0.362] | 0.173 | 0.001 |
| R2 | 0.053 | | | 0.081 | | | 0.093 | | |
| Adjusted R2 | 0.049 | | | 0.074 | | | 0.084 | | |
| F | 12.94 | | | 12.151 | | | 10.057 | | |
| DF | 9, 2076 | | | 15, 2070 | | | 21, 2064 | | |

**S1 Table Continued.**

|  | **Availability of social support** | | | | | | | | |
| --- | --- | --- | --- | --- | --- | --- | --- | --- | --- |
|  | Model 1e | | | Model 2e | | | Model 3e | | |
|  | B [95% Confidence Interval] | β | *p* | B [95% Confidence Interval] | β | *p* | B [95% Confidence Interval] | β | *p* |
| *Covariates* |  |  |  |  |  |  |  |  |  |
| Age | -0.004 [-0.009, 0.002] | -0.027 | 0.231 | -0.001 [-0.007, 0.005] | -0.009 | 0.694 | -0.001 [-0.007, 0.006] | -0.006 | 0.801 |
| Gender (male) | -0.047 [-0.108, 0.015] | -0.033 | 0.135 | -0.053 [-0.114, 0.008] | -0.038 | 0.090 | -0.053 [-0.114, 0.008] | -0.038 | 0.090 |
| Ethnicity (Han) | -0.017 [-0.194, 0.160] | -0.004 | 0.850 | 0.005 [-0.171, 0.181] | 0.001 | 0.958 | 0.008 [-0.168, 0.184] | 0.002 | 0.932 |
| Marital status (married with spouse) | -0.003 [-0.096, 0.090] | -0.002 | 0.946 | -0.005 [-0.098, 0.087] | -0.003 | 0.908 | -0.005 [-0.097, 0.088] | -0.002 | 0.922 |
| Living arrangement (alone) | -0.097 [-0.234, 0.041] | -0.034 | 0.168 | -0.056 [-0.193, 0.081] | -0.020 | 0.424 | -0.056 [-0.193, 0.081] | -0.020 | 0.420 |
| Residence (suburban) | -0.005 [-0.122, 0.113] | -0.002 | 0.938 | 0.010 [-0.107, 0.126] | 0.004 | 0.870 | 0.008 [-0.109, 0.124] | 0.003 | 0.897 |
| Residence (rural) | -0.151 [-0.222, -0.080] | -0.093 | < 0.001 | -0.077 [-0.152, -0.002] | -0.047 | 0.044 | -0.078 [-0.153, -0.003] | -0.048 | 0.042 |
| Self-reported health status | -0.014 [-0.050, 0.021] | -0.018 | 0.424 | -0.020 [-0.056, 0.015] | -0.025 | 0.265 | -0.022 [-0.057, 0.014] | -0.027 | 0.229 |
| Needs for health center services | 0.020 [0.006, 0.340] | 0.061 | 0.005 | 0.017 [0.003, 0.031] | 0.053 | 0.015 | 0.018 [0.004, 0.032] | 0.054 | 0.013 |
| *Socioeconomic status* |  |  |  |  |  |  |  |  |  |
| Education (primary school) |  |  |  | 0.346 [0.218, 0.473] | 0.217 | < 0.001 | 0.338 [0.199, 0.477] | 0.212 | < 0.001 |
| Education (junior high school) |  |  |  | 0.429 [0.302, 0.556] | 0.302 | < 0.001 | 0.417 [0.275, 0.559] | 0.293 | < 0.001 |
| Education (high school) |  |  |  | 0.465 [0.324, 0.605] | 0.256 | < 0.001 | 0.382 [0.216, 0.548] | 0.211 | < 0.001 |
| Education (junior college) |  |  |  | 0.307 [0.127, 0.487] | 0.096 | 0.001 | 0.372[0.171, 0.572] | 0.116 | < 0.001 |
| Education (bachelor’s degree and above) |  |  |  | 0.537 [0.217, 0.858] | 0.076 | 0.001 | 0.530 [0.205, 0.854] | 0.075 | 0.001 |
| Monthly household income |  |  |  | -0.0004 [-0.001, 0.0003] | -0.026 | 0.234 | -0.0005 [-0.002, 0.001] | -0.034 | 0.530 |
| *Interaction terms* |  |  |  |  |  |  |  |  |  |
| Age group × Education (primary school) |  |  |  |  |  |  | -0.013 [-0.091, 0.066] | -0.013 | 0.747 |
| Age group × Education (junior high school) |  |  |  |  |  |  | -0.015 [-0.083, 0.053] | -0.019 | 0.668 |
| Age group × Education (high school) |  |  |  |  |  |  | -0.044 [-0.096, 0.008] | -0.068 | 0.100 |
| Age group × Education (junior college) |  |  |  |  |  |  | 0.020 [-0.010, 0.051] | 0.039 | 0.194 |
| Age group × Education (bachelor’s degree and above) |  |  |  |  |  |  | -0.003 [-0.021, 0.015] | -0.008 | 0.747 |
| Age group × Monthly household income |  |  |  |  |  |  | -0.009 [-0.124, 0.107] | -0.008 | 0.881 |
| R2 | 0.017 | | | 0.041 | | | 0.045 | | |
| Adjusted R2 | 0.013 | | | 0.035 | | | 0.035 | | |
| F | 4.068 | | | 5.972 | | | 4.638 | | |
| DF | 9, 2076 | | | 15, 2070 | | | 21, 2064 | | |
