# Supplementary material for: Socioeconomic differences in digital inequality among Chinese older adults: Results from a nationally representative sample
Source: PLoS One. 2024 Apr 2;19(4):e0300433. doi: 10.1371/journal.pone.0300433 (PMC10986962; doi:10.1371/journal.pone.0300433)
Supplement: S2 Table — (DOCX) [file pone.0300433.s002.docx]

**S2 Table. Results of hierarchical multiple regression for young-old adults.**

|  | **Internet access** | | | | | |
| --- | --- | --- | --- | --- | --- | --- |
|  | Model 1a | | | Model 2a | | |
|  | B [95% Confidence Interval] | β | *p* | B [95% Confidence Interval] | β | *p* |
| *Covariates* |  |  |  |  |  |  |
| Age | -0.020 [-0.023, -0.017] | -0.152 | < 0.001 | -0.016 [-0.019, -0.014] | -0.123 | < 0.001 |
| Gender (male) | -0.011 [-0.032, 0.010] | -0.011 | 0.302 | -0.036 [-0.057, -0.016] | -0.036 | 0.001 |
| Ethnicity (Han) | -0.024 [-0.072, 0.024] | -0.010 | 0.327 | -0.031 [-0.078, 0.016] | -0.014 | 0.192 |
| Marital status (married with spouse) | -0.039 [-0.068, -0.010] | -0.032 | 0.009 | -0.056 [-0.084, -0.027] | -0.047 | < 0.001 |
| Living arrangement (alone) | -0.196 [-0.236, -0.156] | -0.116 | < 0.001 | -0.187 [-0.226, -0.147] | -0.110 | < 0.001 |
| Residence (suburban) | -0.141 [-0.179, -0.103] | -0.080 | < 0.001 | -0.095 [-0.132, -0.057] | -0.054 | < 0.001 |
| Residence (rural) | -0.268 [-0.290, -0.247] | -0.270 | < 0.001 | -0.189 [-0.212, -0.167] | -0.190 | < 0.001 |
| Self-reported health status | 0.033 [0.021, 0.044] | 0.058 | < 0.001 | 0.023 [0.011, 0.035] | 0.041 | < 0.001 |
| Needs for health center services | 0.020 [0.014, 0.025] | 0.075 | < 0.001 | 0.017 [0.012, 0.022] | 0.066 | < 0.001 |
| *Socioeconomic status* |  |  |  |  |  |  |
| Education (primary school) |  |  |  | 0.095 [0.068, 0.122] | 0.095 | < 0.001 |
| Education (junior high school) |  |  |  | 0.220 [0.189, 0.251] | 0.197 | < 0.001 |
| Education (high school) |  |  |  | 0.292 [0.248, 0.335] | 0.163 | < 0.001 |
| Education (junior college) |  |  |  | 0.413 [0.332, 0.494] | 0.108 | < 0.001 |
| Education (bachelor’s degree and above) |  |  |  | 0.461 [0.260, 0.661] | 0.047 | < 0.001 |
| Monthly household income |  |  |  | 0.0006 [0.0004, 0.0008] | 0.065 | < 0.001 |
| R2 | 0.124 | | | 0.164 | | |
| Adjusted R2 | 0.123 | | | 0.162 | | |
| F | 124.246 | | | 103.314 | | |
| DF | 9, 7932 | | | 15, 7926 | | |

**S2 Table Continued.**

|  | **Frequency of Internet use** | | | | | |
| --- | --- | --- | --- | --- | --- | --- |
|  | Model 1b | | | Model 2b | | |
|  | B [95% Confidence Interval] | β | *p* | B [95% Confidence Interval] | β | *p* |
| *Covariates* |  |  |  |  |  |  |
| Age | -0.098 [-0.106, -0.089] | -0.231 | < 0.001 | -0.079 [-0.087, -0.070] | -0.186 | < 0.001 |
| Gender (male) | 0.060 [-0.005, 0.126] | 0.019 | 0.071 | -0.040 [-0.103, 0.023] | -0.013 | 0.210 |
| Ethnicity (Han) | 0.067 [-0.082, 0.217] | 0.009 | 0.379 | 0.055 [-0.088, 0.198] | 0.008 | 0.453 |
| Marital status (married with spouse) | 0.129 [0.038, 0.220] | 0.034 | 0.006 | 0.060 [-0.027, 0.147] | 0.016 | 0.176 |
| Living arrangement (alone) | -0.158 [-0.284, -0.032] | -0.029 | 0.014 | -0.122 [-0.242, -0.002] | -0.023 | 0.047 |
| Residence (suburban) | -0.550 [-0.668, -0.432] | -0.099 | < 0.001 | -0.351 [-0.465, -0.237] | -0.063 | < 0.001 |
| Residence (rural) | -0.806 [-0.874, -0.737] | -0.255 | < 0.001 | -0.476 [-0.546, -0.406] | -0.151 | < 0.001 |
| Self-reported health status | 0.088 [0.051, 0.126] | 0.049 | < 0.001 | 0.044 [0.009, 0.080] | 0.025 | 0.014 |
| Needs for health center services | 0.057 [0.040, 0.073] | 0.069 | < 0.001 | 0.046 [0.030, 0.062] | 0.057 | < 0.001 |
| *Socioeconomic status* |  |  |  |  |  |  |
| Education (primary school) |  |  |  | 0.187 [0.105, 0.270] | 0.059 | < 0.001 |
| Education (junior high school) |  |  |  | 0.838 [0.743, 0.932] | 0.236 | < 0.001 |
| Education (high school) |  |  |  | 1.355 [1.223, 1.487] | 0.238 | < 0.001 |
| Education (junior college) |  |  |  | 1.718 [1.468, 1.967] | 0.141 | < 0.001 |
| Education (bachelor’s degree and above) |  |  |  | 2.231 [1.615, 2.847] | 0.071 | < 0.001 |
| Monthly household income |  |  |  | 0.001 [0.0004, 0.002] | 0.034 | 0.001 |
| R2 | 0.142 | | | 0.217 | | |
| Adjusted R2 | 0.141 | | | 0.216 | | |
| F | 145.475 | | | 146.464 | | |
| DF | 9, 7932 | | | 15, 7926 | | |

**S2 Table Continued.**

|  | **Breadth of Internet use** | | | | | |
| --- | --- | --- | --- | --- | --- | --- |
|  | Model 1c | | | Model 2c | | |
|  | B [95% Confidence Interval] | β | *p* | B [95% Confidence Interval] | β | *p* |
| *Covariates* |  |  |  |  |  |  |
| Age | -0.094 [-0.116, -0.071] | -0.184 | < 0.001 | -0.089 [-0.112, -0.067] | -0.175 | < 0.001 |
| Gender (male) | 0.341 [0.184, 0.497] | 0.096 | < 0.001 | 0.309 [0.153, 0.465] | 0.087 | < 0.001 |
| Ethnicity (Han) | 0.350 [-0.094, 0.795] | 0.034 | 0.122 | 0.425 [-0.016, 0.866] | 0.042 | 0.059 |
| Marital status (married with spouse) | 0.056 [-0.194, 0.306] | 0.011 | 0.660 | 0.019 [-0.228, 0.267] | 0.004 | 0.878 |
| Living arrangement (alone) | 0.259 [-0.111, 0.628] | 0.035 | 0.170 | 0.299 [-0.068, 0.666] | 0.040 | 0.110 |
| Residence (suburban) | -0.162 [-0.460, 0.135] | -0.024 | 0.284 | -0.100 [-0.395, 0.194] | -0.015 | 0.504 |
| Residence (rural) | -0.693 [-0.875, -0.510] | -0.168 | < 0.001 | -0.509 [-0.701, -0.316] | -0.123 | < 0.001 |
| Self-reported health status | 0.064 [-0.027, 0.155] | 0.031 | 0.168 | 0.031 [-0.060, 0.123] | 0.015 | 0.500 |
| Needs for health center services | 0.063 [0.027, 0.099] | 0.077 | 0.001 | 0.061 [0.025, 0.097] | 0.075 | 0.001 |
| *Socioeconomic status* |  |  |  |  |  |  |
| Education (primary school) |  |  |  | 0.922 [0.583, 1.260] | 0.227 | < 0.001 |
| Education (junior high school) |  |  |  | 0.853 [0.519, 1.187] | 0.239 | < 0.001 |
| Education (high school) |  |  |  | 1.017 [0.651, 1.384] | 0.225 | < 0.001 |
| Education (junior college) |  |  |  | 1.493 [1.019, 1.967] | 0.182 | < 0.001 |
| Education (bachelor’s degree and above) |  |  |  | 2.149 [1.233, 3.064] | 0.107 | < 0.001 |
| Monthly household income |  |  |  | 0.0006 [-0.001, 0.002] | 0.017 | 0.453 |
| R2 | 0.760 | | | 0.102 | | |
| Adjusted R2 | 0.072 | | | 0.095 | | |
| F | 17.373 | | | 14.243 | | |
| DF | 9, 1894 | | | 15, 1888 | | |

**S2 Table Continued.**

|  | **Digital skills** | | | | | |
| --- | --- | --- | --- | --- | --- | --- |
|  | Model 1d | | | Model 2d | | |
|  | B [95% Confidence Interval] | β | *p* | B [95% Confidence Interval] | β | *p* |
| *Covariates* |  |  |  |  |  |  |
| Age | -0.027 [-0.038, -0.016] | -0.110 | < 0.001 | -0.021 [-0.031, -0.010] | -0.084 | < 0.001 |
| Gender (male) | 0.015 [-0.061, 0.091] | 0.009 | 0.697 | -0.006 [-0.081, 0.069] | -0.003 | 0.882 |
| Ethnicity (Han) | -0.071 [-0.286, 0.143] | -0.015 | 0.514 | -0.062 [-0.274, 0.150] | -0.013 | 0.564 |
| Marital status (married with spouse) | -0.053 [-0.174, 0.067] | -0.022 | 0.385 | -0.066 [-0.185, 0.053] | -0.028 | 0.275 |
| Living arrangement (alone) | -0.137 [-0.316, 0.041] | -0.038 | 0.131 | -0.117 [-0.293, 0.060] | -0.033 | 0.195 |
| Residence (suburban) | -0.108 [-0.251, 0.036] | -0.033 | 0.141 | -0.065 [-0.206, 0.077] | -0.020 | 0.371 |
| Residence (rural) | -0.363 [-0.451, -0.274] | -0.183 | < 0.001 | -0.266 [-0.359, -0.174] | -0.134 | < 0.001 |
| Self-reported health status | 0.067 [0.023, 0.111] | 0.067 | 0.003 | 0.046 [0.002, 0.090] | 0.046 | 0.040 |
| Needs for health center services | 0.040 [0.023, 0.058] | 0.103 | < 0.001 | 0.035 [0.018, 0.053] | 0.090 | < 0.001 |
| *Socioeconomic status* |  |  |  |  |  |  |
| Education (primary school) |  |  |  | -0.170 [-0.333, -0.008] | -0.087 | 0.040 |
| Education (junior high school) |  |  |  | 0.109 [-0.051, 0.270] | 0.064 | 0.183 |
| Education (high school) |  |  |  | 0.213 [0.037, 0.389] | 0.098 | 0.018 |
| Education (junior college) |  |  |  | 0.397 [0.169, 0.625] | 0.101 | 0.001 |
| Education (bachelor’s degree and above) |  |  |  | 0.116 [-0.324, 0.556] | 0.012 | 0.606 |
| Monthly household income |  |  |  | 0.0005 [-0.0003, 0.001] | 0.027 | 0.230 |
| R2 | 0.065 | | | 0.097 | | |
| Adjusted R2 | 0.060 | | | 0.09 | | |
| F | 14.529 | | | 13.532 | | |
| DF | 9, 1894 | | | 15, 1888 | | |

**S2 Table Continued.**

|  | **Availability of social support** | | | | | |
| --- | --- | --- | --- | --- | --- | --- |
|  | Model 1e | | | Model 2e | | |
|  | B [95% Confidence Interval] | β | *p* | B [95% Confidence Interval] | β | *p* |
| *Covariates* |  |  |  |  |  |  |
| Age | 0.002 [-0.009, 0.009] | 0.0001 | 0.990 | 0.003 [-0.006, 0.012] | 0.015 | 0.530 |
| Gender (male) | -0.051 [-0.115, 0.014] | -0.036 | 0.124 | -0.057 [-0.121, 0.007] | -0.040 | 0.082 |
| Ethnicity (Han) | -0.041 [-0.224, 0.141] | -0.010 | 0.656 | -0.021 [-0.202, 0.161] | -0.005 | 0.822 |
| Marital status (married with spouse) | 0.005 [-0.098, 0.107] | 0.002 | 0.928 | 0.005 [-0.097, 0.107] | 0.002 | 0.928 |
| Living arrangement (alone) | -0.120 [-0.272, 0.031] | -0.041 | 0.120 | -0.079 [-0.230, 0.072] | -0.027 | 0.305 |
| Residence (suburban) | -0.022 [-0.144, 0.101] | -0.008 | 0.730 | -0.010 [-0.131, 0.112] | -0.004 | 0.875 |
| Residence (rural) | -0.144 [-0.219, -0.069] | -0.087 | < 0.001 | -0.073 [-0.152, 0.006] | -0.044 | 0.071 |
| Self-reported health status | -0.024 [-0.061, 0.014] | -0.029 | 0.217 | -0.031 [-0.069, 0.007] | -0.038 | 0.105 |
| Needs for health center services | 0.021 [0.006, 0.035] | 0.064 | 0.006 | 0.018 [0.003, 0.033] | 0.055 | 0.016 |
| *Socioeconomic status* |  |  |  |  |  |  |
| Education (primary school) |  |  |  | 0.345 [0.206, 0.484] | 0.213 | < 0.001 |
| Education (junior high school) |  |  |  | 0.429 [0.291, 0.566] | 0.301 | < 0.001 |
| Education (high school) |  |  |  | 0.485 [0.334, 0.636] | 0.269 | < 0.001 |
| Education (junior college) |  |  |  | 0.262 [0.067, 0.457] | 0.080 | 0.009 |
| Education (bachelor’s degree and above) |  |  |  | 0.549 [0.172, 0.925] | 0.069 | 0.004 |
| Monthly household income |  |  |  | -0.0004 [-0.001, 0.0002] | -0.026 | 0.253 |
| R2 | 0.017 | | | 0.042 | | |
| Adjusted R2 | 0.012 | | | 0.034 | | |
| F | 3.63 | | | 5.518 | | |
| DF | 9, 1894 | | | 15, 1888 | | |
