# Supplementary material for: Socioeconomic differences in digital inequality among Chinese older adults: Results from a nationally representative sample
Source: PLoS One. 2024 Apr 2;19(4):e0300433. doi: 10.1371/journal.pone.0300433 (PMC10986962; doi:10.1371/journal.pone.0300433)
Supplement: S3 Table — (DOCX) [file pone.0300433.s003.docx]

**S3 Table. Results of hierarchical multiple regression for old-old adults.**

|  | **Internet access** | | | | | |
| --- | --- | --- | --- | --- | --- | --- |
|  | Model 1a | | | Model 2a | | |
|  | B [95% Confidence Interval] | β | *p* | B [95% Confidence Interval] | β | *p* |
| *Covariates* |  |  |  |  |  |  |
| Age | -0.001 [-0.004, 0.002] | -0.012 | 0.492 | -0.0004 [-0.004, 0.003] | -0.004 | 0.822 |
| Gender (male) | 0.013 [-0.019, 0.044] | 0.014 | 0.427 | -0.008 [-0.040, 0.024] | -0.008 | 0.624 |
| Ethnicity (Han) | -0.123 [-0.190, -0.057] | -0.061 | < 0.001 | -0.124 [-0.190, -0.058] | -0.061 | < 0.001 |
| Marital status (married with spouse) | -0.074 [-0.109, -0.039] | -0.079 | < 0.001 | -0.086 [-0.121, -0.051] | -0.092 | < 0.001 |
| Living arrangement (alone) | -0.205 [-0.247, -0.162] | -0.169 | < 0.001 | -0.200 [-0.242, -0.158] | -0.165 | < 0.001 |
| Residence (suburban) | -0.162 [-0.220, -0.104] | -0.094 | < 0.001 | -0.132 [-0.189, -0.074] | -0.076 | < 0.001 |
| Residence (rural) | -0.209 [-0.241, -0.177] | -0.222 | < 0.001 | -0.157 [-0.191, -0.123] | -0.166 | < 0.001 |
| Self-reported health status | 0.026 [0.009, 0.042] | 0.050 | 0.002 | 0.022 [0.006, 0.038] | 0.043 | 0.009 |
| Needs for health center services | 0.012 [0.004, 0.020] | 0.049 | 0.003 | 0.010 [0.002, 0.018] | 0.042 | 0.011 |
| *Socioeconomic status* |  |  |  |  |  |  |
| Education (primary school) |  |  |  | 0.054 [0.019, 0.089] | 0.056 | 0.002 |
| Education (junior high school) |  |  |  | 0.085 [0.036, 0.134] | 0.064 | 0.001 |
| Education (high school) |  |  |  | 0.137 [0.067, 0.206] | 0.068 | < 0.001 |
| Education (junior college) |  |  |  | 0.310 [0.199, 0.420] | 0.093 | < 0.001 |
| Education (bachelor’s degree and above) |  |  |  | 0.248 [0.012, 0.485] | 0.034 | 0.040 |
| Monthly household income |  |  |  | 0.0009 [0.0005, 0.001] | 0.084 | < 0.001 |
| R2 | 0.077 | | | 0.098 | | |
| Adjusted R2 | 0.075 | | | 0.094 | | |
| F | 32.149 | | | 25.032 | | |
| DF | 9, 3467 | | | 15, 3461 | | |

**S3 Table Continued.**

|  | **Frequency of Internet use** | | | | | |
| --- | --- | --- | --- | --- | --- | --- |
|  | Model 1b | | | Model 2b | | |
|  | B [95% Confidence Interval] | β | *p* | B [95% Confidence Interval] | β | *p* |
| *Covariates* |  |  |  |  |  |  |
| Age | -0.013 [-0.018, -0.070] | -0.082 | < 0.001 | -0.011 [-0.016, -0.005] | -0.069 | < 0.001 |
| Gender (male) | 0.062 [0.012, 0.113] | 0.042 | 0.016 | 0.025 [-0.026, 0.076] | 0.017 | 0.332 |
| Ethnicity (Han) | 0.106 [-0.001, 0.212] | 0.033 | 0.051 | 0.117 [0.012, 0.221] | 0.037 | 0.029 |
| Marital status (married with spouse) | 0.024 [-0.032, 0.081] | 0.017 | 0.397 | 0.003 [-0.053, 0.058] | 0.002 | 0.929 |
| Living arrangement (alone) | -0.065 [-0.133, 0.003] | -0.034 | 0.063 | -0.056 [-0.123, 0.011] | -0.030 | 0.100 |
| Residence (suburban) | -0.133 [-0.226, -0.041] | -0.049 | 0.005 | -0.089 [-0.181, 0.003] | -0.033 | 0.057 |
| Residence (rural) | -0.128 [-0.179, -0.077] | -0.087 | < 0.001 | -0.045 [-0.100, 0.009] | -0.031 | 0.102 |
| Self-reported health status | 0.012 [-0.015, 0.038] | 0.015 | 0.384 | 0.004 [-0.022, 0.030] | 0.005 | 0.770 |
| Needs for health center services | 0.008 [-0.005, 0.020] | 0.021 | 0.222 | 0.005 [-0.007, 0.018] | 0.014 | 0.418 |
| *Socioeconomic status* |  |  |  |  |  |  |
| Education (primary school) |  |  |  | 0.056 [0.001, 0.111] | 0.037 | 0.051 |
| Education (junior high school) |  |  |  | 0.244 [0.166, 0.322] | 0.118 | < 0.001 |
| Education (high school) |  |  |  | 0.270 [0.159, 0.381] | 0.086 | < 0.001 |
| Education (junior college) |  |  |  | 0.535 [0.359, 0.711] | 0.103 | < 0.001 |
| Education (bachelor’s degree and above) |  |  |  | 1.321 [0.944, 1.698] | 0.115 | < 0.001 |
| Monthly household income |  |  |  | 0.0002 [-0.0003, 0.0008] | 0.013 | 0.447 |
| R2 | 0.022 | | | 0.054 | | |
| Adjusted R2 | 0.02 | | | 0.050 | | |
| F | 8.778 | | | 13.217 | | |
| DF | 9, 3467 | | | 15, 3461 | | |

**S3 Table Continued.**

|  | **Breadth of Internet use** | | | | | |
| --- | --- | --- | --- | --- | --- | --- |
|  | Model 1c | | | Model 2c | | |
|  | B [95% Confidence Interval] | β | *p* | B [95% Confidence Interval] | β | *p* |
| *Covariates* |  |  |  |  |  |  |
| Age | -0.017 [-0.062, 0.029] | -0.057 | 0.475 | -0.020 [-0.064, 0.025] | -0.067 | 0.393 |
| Gender (male) | 0.018 [-0.415, 0.451] | 0.007 | 0.935 | -0.018 [-0.447, 0.411] | -0.007 | 0.934 |
| Ethnicity (Han) | 1.638 [0.059, 3.216] | 0.156 | 0.042 | 1.736 [0.131, 3.340] | 0.165 | 0.034 |
| Marital status (married with spouse) | 0.033 [-0.442, 0.508] | 0.012 | 0.892 | 0.049 [-0.427, 0.525] | 0.018 | 0.839 |
| Living arrangement (alone) | 0.158 [-0.516, 0.833] | 0.037 | 0.644 | 0.072 [-0.605, 0.748] | 0.017 | 0.835 |
| Residence (suburban) | 0.221 [-0.667, 1.110] | 0.038 | 0.624 | 0.324 [-0.574, 1.223] | 0.055 | 0.477 |
| Residence (rural) | 0.167 [-0.289, 0.623] | 0.058 | 0.471 | 0.406 [-0.093, 0.906] | 0.141 | 0.110 |
| Self-reported health status | -0.030 [-0.247, 0.187] | -0.021 | 0.786 | -0.047 [-0.274, 0.180] | -0.033 | 0.682 |
| Needs for health center services | 0.026 [-0.071, 0.123] | 0.041 | 0.596 | 0.035 [-0.063, 0.133] | 0.054 | 0.488 |
| *Socioeconomic status* |  |  |  |  |  |  |
| Education (primary school) |  |  |  | 0.168 [-0.501, 0.838] | 0.060 | 0.620 |
| Education (junior high school) |  |  |  | -0.035 [-0.739, 0.669] | -0.012 | 0.921 |
| Education (high school) |  |  |  | 0.081 [-0.782, 0.943] | 0.019 | 0.854 |
| Education (junior college) |  |  |  | 0.198 [-0.788, 1.184] | 0.038 | 0.692 |
| Education (bachelor’s degree and above) |  |  |  | 0.338 [-0.973, 1.648] | 0.045 | 0.612 |
| Monthly household income |  |  |  | 0.002 [0.0006, 0.003] | 0.249 | 0.003 |
| R2 | 0.033 | | | 0.094 | | |
| Adjusted R2 | -0.018 | | | 0.012 | | |
| F | 0.65 | | | 1.143 | | |
| DF | 9, 172 | | | 15, 166 | | |

**S3 Table Continued.**

|  | **Digital skills** | | | | | |
| --- | --- | --- | --- | --- | --- | --- |
|  | Model 1d | | | Model 2d | | |
|  | B [95% Confidence Interval] | β | *p* | B [95% Confidence Interval] | β | *p* |
| *Covariates* |  |  |  |  |  |  |
| Age | 0.025 [-0.001, 0.052] | 0.146 | 0.063 | 0.023 [-0.003, 0.049] | 0.132 | 0.086 |
| Gender (male) | -0.071 [-0.326, 0.185] | -0.043 | 0.585 | -0.098 [-0.349,0.154] | -0.060 | 0.444 |
| Ethnicity (Han) | 1.276 [0.345, 2.207] | 0.202 | 0.007 | 1.235 [0.295, 2.175] | 0.196 | 0.010 |
| Marital status (married with spouse) | -0.030 [-0.311, 0.250] | -0.018 | 0.831 | -0.012 [-0.291, 0.266] | -0.007 | 0.931 |
| Living arrangement (alone) | -0.177 [-0.575, 0.221] | -0.069 | 0.381 | -0.261 [-0.658, 0.135] | -0.102 | 0.195 |
| Residence (suburban) | -0.071 [-0.595, 0.453] | -0.020 | 0.789 | -0.025 [-0.551, 0.502] | -0.007 | 0.927 |
| Residence (rural) | -0.070 [-0.339, 0.199] | -0.040 | 0.610 | 0.043 [-0.249, 0.336] | 0.025 | 0.77 |
| Self-reported health status | -0.018 [-0.146, 0.110] | -0.021 | 0.781 | -0.007 [-0.140, 0.126] | -0.008 | 0.915 |
| Needs for health center services | -0.030 [-0.087, 0.028] | -0.077 | 0.311 | -0.027 [-0.085, 0.030] | -0.071 | 0.346 |
| *Socioeconomic status* |  |  |  |  |  |  |
| Education (primary school) |  |  |  | 0.157 [-0.235, 0.549] | 0.093 | 0.431 |
| Education (junior high school) |  |  |  | 0.036 [-0.377, 0.448] | 0.02 | 0.865 |
| Education (high school) |  |  |  | -0.006 [-0.511, 0.500] | -0.002 | 0.983 |
| Education (junior college) |  |  |  | -0.172 [-0.750, 0.406] | -0.055 | 0.557 |
| Education (bachelor’s degree and above) |  |  |  | -0.157 [-0.925, 0.611] | -0.035 | 0.688 |
| Monthly household income |  |  |  | 0.001 [0.0005, 0.002] | 0.279 | 0.001 |
| R2 | 0.066 | | | 0.136 | | |
| Adjusted R2 | 0.017 | | | 0.058 | | |
| F | 1.348 | | | 1.746 | | |
| DF | 9, 172 | | | 15, 166 | | |

**S3 Table Continued.**

|  | **Availability of social support** | | | | | |
| --- | --- | --- | --- | --- | --- | --- |
|  | Model 1e | | | Model 2e | | |
|  | B [95% Confidence Interval] | β | *p* | B [95% Confidence Interval] | β | *p* |
| *Covariates* |  |  |  |  |  |  |
| Age | -0.0002 [-0.022, 0.022] | -0.002 | 0.983 | 0.001 [-0.021, 0.022] | 0.004 | 0.957 |
| Gender (male) | 0.021 [-0.187, 0.230] | 0.016 | 0.842 | 0.019 [-0.187, 0.225] | 0.014 | 0.857 |
| Ethnicity (Han) | 0.395 [-0.365, 1.155] | 0.077 | 0.306 | 0.547 [-0.223, 1.316] | 0.107 | 0.163 |
| Marital status (married with spouse) | -0.143 [-0.371, 0.086] | -0.106 | 0.220 | -0.153 [-0.381, 0.075] | -0.114 | 0.187 |
| Living arrangement (alone) | 0.013 [-0.311, 0.338] | 0.006 | 0.935 | 0.064 [-0.260, 0.388] | 0.031 | 0.698 |
| Residence (suburban) | 0.196 [-0.231, 0.624] | 0.069 | 0.366 | 0.226 [-0.205, 0.657] | 0.079 | 0.302 |
| Residence (rural) | -0.219 [-0.438, 0.001] | -0.156 | 0.051 | -0.095 [-0.335, 0.145] | -0.067 | 0.436 |
| Self-reported health status | 0.059 [-0.046, 0.163] | 0.084 | 0.268 | 0.056 [-0.053, 0.165] | 0.080 | 0.308 |
| Needs for health center services | 0.004 [-0.043, 0.051] | 0.012 | 0.874 | 0.006 [-0.041, 0.053] | 0.020 | 0.797 |
| *Socioeconomic status* |  |  |  |  |  |  |
| Education (primary school) |  |  |  | 0.410 [0.089, 0.731] | 0.299 | 0.013 |
| Education (junior high school) |  |  |  | 0.466 [0.129, 0.804] | 0.329 | 0.007 |
| Education (high school) |  |  |  | 0.269 [-0.144, 0.683] | 0.132 | 0.201 |
| Education (junior college) |  |  |  | 0.703 [0.230, 1.176] | 0.278 | 0.004 |
| Education (bachelor’s degree and above) |  |  |  | 0.656 [0.027, 1.284] | 0.180 | 0.041 |
| Monthly household income |  |  |  | -0.0008 [-0.006, 0.004] | -0.025 | 0.758 |
| R2 | 0.054 | | | 0.120 | | |
| Adjusted R2 | 0.005 | | | 0.041 | | |
| F | 1.093 | | | 1.512 | | |
| DF | 9, 172 | | | 15, 166 | | |
